# Supplementary figures and images for: The impact of pneumococcal vaccination on pneumonia mortality among the elderly in Japan: a difference-in-difference study
Source: PeerJ. 2018 Dec 12;6:e6085. doi: 10.7717/peerj.6085 (PMC6295158; doi:10.7717/peerj.6085)

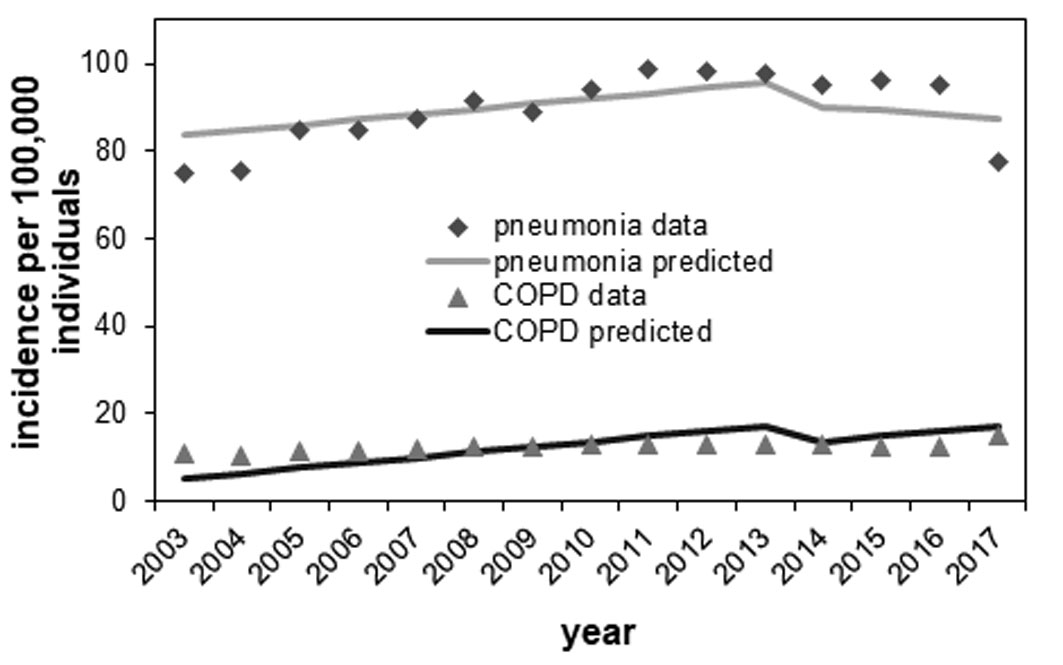

Supplement: Supplemental Information 1 — Comparison of observed and predicted mortality of pneumonia and chronic obstructive pulmonary disease (COPD) from 2003-2017, Japan. Predicted mortality for the entire Japan was calculated, using the COPD mortality as the control group. The vertical axis represents the mortality per 100,000 individuals. Each line represents the predicted mortality, while each mark represents the observed data. [file peerj-06-6085-s001.jpg]
