# Supplementary material for: The impact of pneumococcal vaccination on pneumonia mortality among the elderly in Japan: a difference-in-difference study
Source: PeerJ. 2018 Dec 12;6:e6085. doi: 10.7717/peerj.6085 (PMC6295158; doi:10.7717/peerj.6085)
Supplement: Supplemental Information 2 [file peerj-06-6085-s002.docx]

## Supplementary Table 1. Correlation between pneumonia and control diseases (i.e., malignant neoplasm and heart disease) by age and gender

| Control groups | Age (years) / Gender | From 2003-13 | | From 2014-17 | |
| --- | --- | --- | --- | --- | --- |
|  |  | R^2^ | *P* -value | R^2^ | *P* -value |
| malignant neoplasm | male | 0.82 | < 0.001 | 0.86 | < 0.001 |
|  | female | 0.84 | < 0.001 | 0.86 | < 0.001 |
| heart disease | male | 0.99 | < 0.001 | 0.98 | < 0.001 |
|  | female | 0.99 | < 0.001 | 0.98 | < 0.001 |
| malignant neoplasm | 65-69 | 0.99 | < 0.001 | 0.95 | < 0.001 |
|  | 70-74 | 0.99 | < 0.001 | 0.94 | < 0.001 |
|  | 75-79 | 1.00 | < 0.001 | 0.94 | < 0.001 |
|  | 80-84 | 0.99 | < 0.001 | 0.93 | < 0.001 |
|  | 85-89 | 0.98 | < 0.001 | 0.93 | < 0.001 |
|  | 90 & over | 0.96 | < 0.001 | 0.92 | < 0.001 |
| heart disease | 65-69 | 0.99 | < 0.001 | 0.94 | < 0.001 |
|  | 70-74 | 0.98 | < 0.001 | 0.95 | < 0.001 |
|  | 75-79 | 0.98 | < 0.001 | 0.94 | < 0.001 |
|  | 80-84 | 0.97 | < 0.001 | 0.95 | < 0.001 |
|  | 85-89 | 0.93 | < 0.001 | 0.93 | < 0.001 |
|  | 90 & over | 0.73 | < 0.001 | 0.90 | < 0.001 |

Correlation between mortality of pneumonia and control groups by age and gender are shown (by means of linear regression analysis). Malignant neoplasm and heart disease were used as control groups.
