# Supplementary material for: The impact of pneumococcal vaccination on pneumonia mortality among the elderly in Japan: a difference-in-difference study
Source: PeerJ. 2018 Dec 12;6:e6085. doi: 10.7717/peerj.6085 (PMC6295158; doi:10.7717/peerj.6085)
Supplement: Supplemental Information 3 [file peerj-06-6085-s003.docx]

## Supplementary Table 2. Summary of causal model parameters for the analysis by age-group and gender

|  |  | With 2017 | | | | Without 2017 | | | |
| --- | --- | --- | --- | --- | --- | --- | --- | --- | --- |
|  |  | malignant neoplasm | | heart disease | | malignant neoplasm | | heart disease | |
|  |  | Male | Female | Male | Female | Male | Female | Male | Female |
| 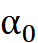 | 65-69 | 725  (652, 798) | 306  (274, 339) | 206  (141, 272) | 77  (36, 118) | 725  (650, 800) | 306  (272, 340) | 206  (141, 272) | 78  (37, 118) |
|  | 70-74 | 1146  (1073, 1219) | 450  (417, 484) | 340 (275, 406) | 160  (119, 202) | 1147 (1072, 1221) | 450  (416, 484) | 341  (275, 406) | 161  (120, 201) |
|  | 75-79 | 1673  (1600, 1746) | 655  (622, 688) | 599  (533, 664) | 339  (298, 380) | 1673 (1599, 1748) | 654  (620, 689) | 599  (534, 665) | 339  (298, 380) |
|  | 80-84 | 2253  (2180, 2326) | 961  (928, 995) | 1073 (1007, 1138) | 739  (698, 781) | 2252 (2178, 2327) | 961  (927, 996) | 1073 (1008, 1139 ) | 740  (699, 781) |
|  | 85-89 | 2968  (2895, 3041) | 1402 (1368, 1435) | 2043 (1977, 2108) | 1587 (1546, 1628) | 2969 (2894, 3043) | 1403 (1368, 1437) | 2047 (1981, 2112) | 1589 (1548, 1629) |
|  | 90 & over | 3467  (3394, 3540.26) | 1760 (1727, 1794) | 3804 (3739, 3870) | 3417 (3376, 3458) | 3470 (3395, 3545) | 1762 (1728, 1797 ) | 3809 (3744, 3875 ) | 3418 (3377, 3459) |
| 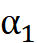 | 65-69 | -6  (-16, 5) | -1  (-6, 4) | -2  (-12, 8) | -2  (-8, 5) | -5  (-17, 6) | -1  (-6, 4) | -2  (-12, 8) | -1  (-8, 5) |
|  | 70-74 | -16  (-27, -6) | -4  (-9, 01) | -6  (-16, 3) | -4  (-10, 2) | -16  (-27, -5) | -4  (-9, 1) | -6  (-16, 4) | -4  (-10, 3) |
|  | 75-79 | -19  (-30, -8) | -6  (-10, -1) | -11  (-21, -1) | -7  (-13, -1) | -18  (-30, -7) | -5  (-10, -0) | -11  (-21, -1) | -7  (-13, -1) |
|  | 80-84 | -13  (-23, -2) | -8  (-13, -3) | -14  (-24, -4) | -13  (-19, -7) | -12  (-23, 0) | -8  (-13, -3) | -13  (-23, -4) | -12  (-19, -6) |
|  | 85-89 | -22  (-33, -11) | -16 (-21, -11) | -32  (-42, -22) | -26  (-32, -20) | -22  (-33, -11) | -16  (-21, -11 ) | -32  (-41, -22) | -25  (-31, -19) |
|  | 90 & over | -5  (-16, 6) | -11  (-16, -6) | -12  (-22, -3) | -7  (-13, 0) | -5  (-16, 6) | -11  (-16, -6) | -12  (-22, -3) | -6  (-12, 0) |
| 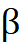 | 65-69 | 16  (-28, 59) | 1  (-19, 21) | 2  (-37, 41) | 3  (-22, 27) | 22  (-42, 86) | 3  (-27, 32) | 6  (-50, 63) | 5  (-30, 40) |
|  | 70-74 | 23  (-20, 67) | 5  (-15, 24) | 2  (-37, 41) | 5  (-19, 30) | 34  (-30, 98) | 8  (-22, 37) | 9  (-47, 66) | 9  (-26, 44) |
|  | 75-79 | 32  (-11, 75) | 3  (-16, 23) | -9  (-48, 30) | 8  (-16, 33) | 53  (-11, 117) | 8  (-21, 37) | 4  (-53, 60) | 16  (-19, 51) |
|  | 80-84 | -31  (-74, 12) | -18  (-38, 2) | -42  (-81, -3) | 7  (-18, 31) | -8  (-72, 56) | -11  (-41, 18) | -20  (-76, 36) | 21  (-14, 56) |
|  | 85-89 | -143  (-186, -99) | -60  (-80, -40) | -98  (-137, -59) | -12  (-37, 12) | -116  (-180, -52) | -49  (-79, -20) | -52  (-108, 4) | 12  (-23, 47) |
|  | 90 & over | -357  (-401,-314) | -199  (-218, -179) | -276  (-315, -237) | -137  (-162, -113) | -339  (-403, -275) | -186  (-215, -156) | -226  (-282, -170) | -105  (-140, -70) |
| 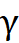 | 65-69 | -628  (-694,-562) | -279  (-309, -249) | -128  (-187, -69) | -49  (-87, -12) | -630  (-698, -561) | -279  (-310, -248 ) | -129  (-189, -69 ) | -50  (-87, -12) |
|  | 70-74 | -905  (-971, -839) | -379  (-409, -349) | -151  (-211, -92) | -92  (-129, -55) | -908  (-977, -840) | -380  (-411, -349) | -153  (-213, -93) | -93  (-130, -56) |
|  | 75-79 | -1183  (-1249, -1117) | -496  (-526, -466) | -155  (-214, -96) | -173  (-210, -136) | -1191  (-1259, -1122) | -498  (-529, -467 ) | -158  (-218, -98) | -175  (-213, -138) |
|  | 80-84 | -1271  (-1337, -1205) | -573  (-603, -543) | -86  (-145, -27) | -324  (-361, -287) | -1280  (-1348, -1212) | -575  (-607, -544) | -93  (-153, -33) | -329  (-366, -291) |
|  | 85-89 | -869  (-935, -803) | -452  (-482, -422) | 112  (53, 171) | -582  (-619, -545) | -876  (-945, -808) | -454  (-485, -423 ) | 102  (42, 162 ) | -589  (-626, -551) |
|  | 90 & over | 955  (889, 1021) | 618  (588, 648) | 668  (609, 727) | -1049  (-1086, -1012) | 952  (884, 1021 | 616  (585, 648 ) | 658  (598, 718) | -1059  (-1096, -1022) |
| δ | 65-69 | -19  (-134, 96) | 2  (-50, 54) | -5  (-109, 98) | -3  (-68, 61) | -22  (-146, 102) | 0  (-57, 57 ) | -9  (-118, 100 ) | -5  (-73, 63 ) |
|  | 70-74 | 17  (-98, 132) | 4  (-49, 56) | 2  (-101, 105) | -4  (-68, 61) | 10  (-114, 135) | 2  (-54, 59 ) | -3  (-112, 106 ) | -5  (-73, 62) |
|  | 75-79 | -75  (-190, 40) | -11  (-63, 41) | -12  (-115, 91) | -16  (-81, 49) | -85  (-209, 39) | -11  (-68, 46 ) | -20  (-129, 89 ) | -19  (-87, 49) |
|  | 80-84 | -89  (-204, 25) | 5  (-48, 57) | -41  (-144, 62) | -36  (-101, 29) | -96  (-221, 28) | 1  (-56, 58 ) | -54  (-163, 55 ) | -44  (-112, 24) |
|  | 85-89 | 79  (-36, 194) | 30  (-22, 82) | 18  (-86, 121) | -43 (-108, 22) | 63  (-61, 187) | 21  (-36, 78 ) | -17  (-126, 92 ) | -59  (-127, 9 ) |
|  | 90 & over | -8  (-122, 107) | 28  (-25, 80) | -206  (-309, -103) | -204  (-269, -140) | -27  (-151, 98) | 13  (-44, 70 ) | -247.74  (-357, -139 ) | -222  (-290, -154) |

Malignant neoplasm and heart disease were used as control groups. Upper and lower 95% confidence intervals, derived from profile likelihood, are shown in parenthesis.
