# Supplementary material for: The impact of pneumococcal vaccination on pneumonia mortality among the elderly in Japan: a difference-in-difference study
Source: PeerJ. 2018 Dec 12;6:e6085. doi: 10.7717/peerj.6085 (PMC6295158; doi:10.7717/peerj.6085)
Supplement: Supplemental Information 4 [file peerj-06-6085-s004.docx]

## Supplementary Table 3. Summary of observed and predicted mortality from the analysis of prefectural data

| Year | Predicted | | | Observed | | |
| --- | --- | --- | --- | --- | --- | --- |
|  |  |  |  |  |  |  |
|  | malignant neoplasm | heart disease | pneumonia | malignant neoplasm | heart disease | pneumonia |
| 2003 | 267 | 141 | 81 | 257  (173, 331) | 139  (91, 176) | 83  (56, 114) |
| 2004 | 271 | 145 | 85 | 266  (186, 329) | 139  (87, 182) | 82  (56, 119) |
| 2005 | 275 | 148 | 88 | 272  (185, 337) | 153  (92, 198) | 96  (64, 125) |
| 2006 | 279 | 151 | 92 | 277  (185, 343) | 152  (95, 200) | 94  (61, 132) |
| 2007 | 282 | 155 | 96 | 282  (191, 352) | 153  (96, 201) | 96  (61, 132) |
| 2008 | 286 | 158 | 100 | 287  (198, 355) | 158  (100, 210) | 101  (66, 140) |
| 2009 | 290 | 161 | 103 | 287  (192, 367) | 159  (104, 207) | 99  (65, 138) |
| 2010 | 294 | 165 | 107 | 293  (198, 377) | 164  (105, 223) | 103  (65, 146) |
| 2011 | 297 | 168 | 111 | 299  (204, 377) | 173  (110, 226) | 112  (67, 152) |
| 2012 | 301 | 171 | 115 | 302  (208, 387) | 177  (112, 249) | 106  (66, 157) |
| 2013 | 305 | 175 | 118 | 301  (213, 393) | 174  (108, 245) | 107  (71, 161) |
| 2014 | 309 | 173 | 112 | 308  (210, 407) | 175  (112, 240) | 104  (64, 158) |
| 2015 | 313 | 177 | 106 | 306  (219, 408) | 175  (109, 240) | 106  (63, 159) |
| 2016 | 317 | 180 | 99 | 309  (215, 421) | 178  (113, 245) | 101  (61, 163) |
| 2017 | 321 | 183 | 92 | 313  (212, 413) | 181  (116, 254) | 84  (48, 134) |

Mortality per 100,000 individuals are shown. Malignant neoplasm and heart disease were used as control groups. Observed data represent median value, while in parenthesis, mean and maximum values are given.
