# Supplementary material for: The impact of pneumococcal vaccination on pneumonia mortality among the elderly in Japan: a difference-in-difference study
Source: PeerJ. 2018 Dec 12;6:e6085. doi: 10.7717/peerj.6085 (PMC6295158; doi:10.7717/peerj.6085)
Supplement: Supplemental Information 5 — Supplementary Table 4. Estimates of the regression parameters of the causal effect model, comparing pneumonia mortality with that of chronic obstructive pulmonary disease (COPD) in Japan. [file peerj-06-6085-s005.docx]

## Supplementary Table 4. Estimates of the regression parameters of the causal effect model, comparing pneumonia mortality with that of chronic obstructive pulmonary disease (COPD) in Japan

| Parameters | Estimates | Lower 95% CI | Upper 95% CI |
| --- | --- | --- | --- |
| $\alpha_{0}$ | 5 | 1 | 10 |
| $\alpha_{1}$ | 1 | 1 | 2 |
| $\beta$ | -2 | -5 | 1 |
| $\gamma$ | 78 | 74 | 82 |
| $\delta$ | -5 | -12 | 2 |

Figures should be interpreted as the yearly rate of reduction in pneumonia mortality per 100,000 individuals. All estimates are derived from the entire country data using model (1) with a COPD as a control group. The datasets in 2017 were included. Upper and lower 95% confidence intervals (CI) were derived from the profile likelihood.
